# Persistent severe acute respiratory distress syndrome for the prognostic enrichment of trials

**DOI:** 10.1371/journal.pone.0227346

**Published:** 2020-01-27

**Authors:** Elizabeth Sanchez, David R. Price, Kuei-Pin Chung, Clara Oromendia, Augustine M. K. Choi, Edward J. Schenck, Ilias I. Siempos

**Affiliations:** 1 Division of Pulmonary and Critical Care Medicine, Department of Medicine, New York-Presbyterian Hospital-Weill Cornell Medical Center, Weill Cornell Medicine, New York, New York, United States of America; 2 Department of Laboratory Medicine, National Taiwan University Hospital, Taipei, Taiwan; 3 Division of Biostatistics and Epidemiology, Department of Healthcare Policy and Research, Weill Cornell Medicine, New York, New York, United States of America; 4 First Department of Critical Care Medicine and Pulmonary Services, Evangelismos Hospital, University of Athens Medical School, Athens, Greece; Universite de Bretagne Occidentale, FRANCE

## Abstract

**Background:**

Acute respiratory distress syndrome (ARDS) is heterogeneous. As an indication of the heterogeneity of ARDS, there are patients whose syndrome improves rapidly (i.e., within 24 hours), others whose hypoxemia improves gradually and still others whose severe hypoxemia persists for several days. The latter group of patients with persistent severe ARDS poses challenges to clinicians. We attempted to assess the baseline characteristics and outcomes of persistent severe ARDS and to identify which variables are useful to predict it.

**Methods:**

A secondary analysis of patient-level data from the ALTA, EDEN and SAILS ARDSNet clinical trials was conducted. We defined persistent severe ARDS as a partial pressure of arterial oxygen to fraction of inspired oxygen ratio (PaO_2_:FiO_2_) of equal to or less than 100 mmHg on the second study day following enrollment. Regularized logistic regression with an L1 penalty [Least Absolute Shrinkage and Selection Operator (LASSO)] techniques were used to identify predictive variables of persistent severe ARDS.

**Results:**

Of the 1531 individuals with ARDS alive on the second study day after enrollment, 232 (15%) had persistent severe ARDS. Of the latter, 100 (43%) individuals had mild or moderate hypoxemia at baseline. Usage of vasopressors was greater [144/232 (62%) versus 623/1299 (48%); p<0.001] and baseline severity of illness was higher in patients with versus without persistent severe ARDS. Mortality at 60 days [95/232 (41%) versus 233/1299 (18%); p<0.001] was higher, and ventilator-free (p<0.001), intensive care unit-free [0 (0–14) versus 19 (7–23); p<0.001] and non-pulmonary organ failure-free [3 (0–21) versus 20 (1–26); p<0.001] days were fewer in patients with versus without persistent severe ARDS. PaO_2_:FiO_2_, FiO_2_, hepatic failure and positive end-expiratory pressure at enrollment were useful predictive variables.

**Conclusions:**

Patients with persistent severe ARDS have distinct baseline characteristics and poor prognosis. Identifying such patients at enrollment may be useful for the prognostic enrichment of trials.

## Introduction

Acute respiratory distress syndrome (ARDS) is heterogeneous [1–3]. As an indication of the heterogeneity of ARDS, there are patients whose syndrome improves rapidly (i.e., within 24 hours) [4, 5], others whose hypoxemia improves gradually and still others whose severe hypoxemia persists for several days [6]. The latter group of patients with persistent severe ARDS may be the most challenging to clinicians.

From a research perspective, identification and subsequent enrollment of patients with persistent severe ARDS into therapeutic trials may theoretically be used for prognostic and/or predictive enrichment of such trials. Prognostic enrichment refers to selective enrollment of patients who are likely to experience the outcome of interest (such as mortality) so that the sample size needed to reveal a statistically significant treatment effect can be reduced [1]. Predictive enrichment refers to selective enrollment of patients who are likely to respond to treatment because they share a common underlying biology and histopathology [1]. To this point, at post-mortem lung examination, patients with persistent severe ARDS often share the landmark histopathological feature of ARDS, namely diffuse alveolar damage [7].

Having the above considerations in mind, we sought to explore the baseline characteristics and assess the outcomes of patients with persistent severe ARDS. We hypothesized that patients with as opposed to those without persistent severe ARDS have distinct baseline characteristics, worse clinical outcomes (making them appropriate for prognostic enrichment) and different response to treatment (making them appropriate for predictive enrichment). We also attempted to identify which variables may be useful to predict persistent severe ARDS.

## Methods

### Study design and patient population

This was a secondary analysis of patient-level data from randomized controlled trials obtained through the Biologic Specimen and Data Repository Information Coordinating Center (BioLINCC) of National Institutes of Health-National Heart, Lung, and Blood Institute (NIH-NHLBI) [8]. Data from the three most recently published ARDSNet trials, namely ALTA (comparing aerosolized albuterol versus placebo), EDEN (initial trophic versus full enteral feeding) and SAILS (rosuvastatin versus placebo) were analyzed [9–11]. These trials were published after 2010 and therefore were expected to reflect modern clinical practice in the intensive care unit (ICU) [9–11]. Details of these trials, including inclusion criteria, have been previously published [9–11]. Briefly, all enrolled patients were endotracheally intubated undergoing positive pressure mechanical ventilation, had a partial pressure of arterial oxygen to fraction of inspired oxygen ratio (PaO_2_:FiO_2_) of 300 mmHg or less, and had bilateral infiltrates on chest radiography consistent with non-cardiogenic pulmonary edema [9–11]. The Institutional Review Board at Weill Cornell Medicine approved of this secondary analysis (#1709018558). The need for informed consent was waived.

### Definition of persistent severe ARDS

The group of patients with persistent severe ARDS comprised of endotracheally intubated individuals receiving positive pressure ventilation and having a PaO_2_:FiO_2_ of equal to or less than 100 mmHg on the second study day after trial enrollment. The group of patients without persistent severe ARDS comprised of individuals who were alive on the second study day after trial enrollment, but they were not endotracheally intubated or they had a PaO_2_:FiO_2_ of more than 100 mmHg. A similar definition of persistent severe ARDS was recently used by the Lung Safe investigators [12]. We also carried out a sensitivity analysis by defining persistent severe ARDS as PaO_2_:FiO_2_ of equal to or less than 100 mmHg on the third (rather than second) study day. The rationale behind this sensitivity analysis was the previous observation that diffuse alveolar damage is frequent in patients meeting clinical criteria of ARDS for at least 72 hours [7].

### Outcomes

All cause 60-day mortality between patients with versus without persistent severe ARDS was the primary outcome of this secondary analysis. Patients discharged from hospital with unassisted breathing were considered alive at 60 days. Ventilator-free days, ICU-free days and non-pulmonary organ failure-free days in the first 28 days were the secondary outcomes. Both the primary and secondary outcomes were compared across experimental groups of each individual trial [9–11] among patients with versus without persistent severe ARDS.

### Statistical analyses and identification of predictive variables

Statistical analyses were done with R v3.2.3 (R Core Team, Vienna, Austria). A two-tailed p value of less than 0.05 was considered statistically significant. Continuous and categorical variables were presented for patients with versus without persistent severe ARDS using medians (interquartile range) and count (percentages), respectively. Differences between the two groups were tested using non-parametric Mann-Whitney U test and chi-square test, respectively.

A predictive model was created using baseline characteristics to identify patients at high risk of having severe ARDS (i.e., PaO_2_:FiO_2_ of equal to or less than 100 mmHg) on the second study day after trial enrollment. Patients who died within the first two study days after trial enrollment (i.e., a total of 56 patients; 37 in the derivation and 19 in the validation cohort) were included in the predictive model with the rationale being that both groups of patients (i.e., those at risk of refractory hypoxemia and those at risk of early death) require the immediate attention of caregivers and the potential usage of aggressive treatment. Given the detailed characterization of this population, more than 70 features were available to be used, and machine learning techniques were required. Patients were randomly divided into a derivation set (66%) and a validation set (33%) in order to test internal validity of our chosen model. Using the derivation dataset only, and selecting variables which were available in at least 85% of patients, we explored several techniques to identify the characteristics most important for prediction that led to a parsimonious regression model. These techniques included traditional stepwise AIC-based procedures, regularized regressions, and clinically chosen models, fit on all predictors as well as a subset. This subset of predictors was selected using random forests, an ensemble classification modeling technique, to order variables by Gini importance [13], and the top 20 variables were selected. Then, this subset of predictors was used in Least Absolute Shrinkage and Selection Operator (LASSO) logistic regression, an elastic net regularization method that finds a parsimonious logistic regression [14]. This was a logistic regression predicting severe ARDS on the second study day after trial enrollment, using only the variables that were present in the top 20 importance ranking in the random forest analysis as well as the LASSO regression. Multicollinearity of the model was explored using correlation and variance inflation factor (VIF), with a VIF greater than 2 considered problematic. Accuracy of the model was measured with the area under the receiver operating curves (AUC), then predictions were dichotomized at the Youden’s optimal cut point and sensitivity, specificity, and negative and positive predicted values were calculated, with 95% confidence intervals (CI) for each. This same model was then used to predict outcomes in the validation dataset, to this point unused. AUC, sensitivity, specificity, and negative and positive predicted values were again used to measure accuracy. A similar technique was used to identify patients with mild or moderate hypoxemia at trial enrollment who deteriorated to severe hypoxemia (i.e., PaO_2_:FiO_2_ of equal to or less than 100 mmHg) on the second study day after trial enrollment.

## Results

**Fig 1:**
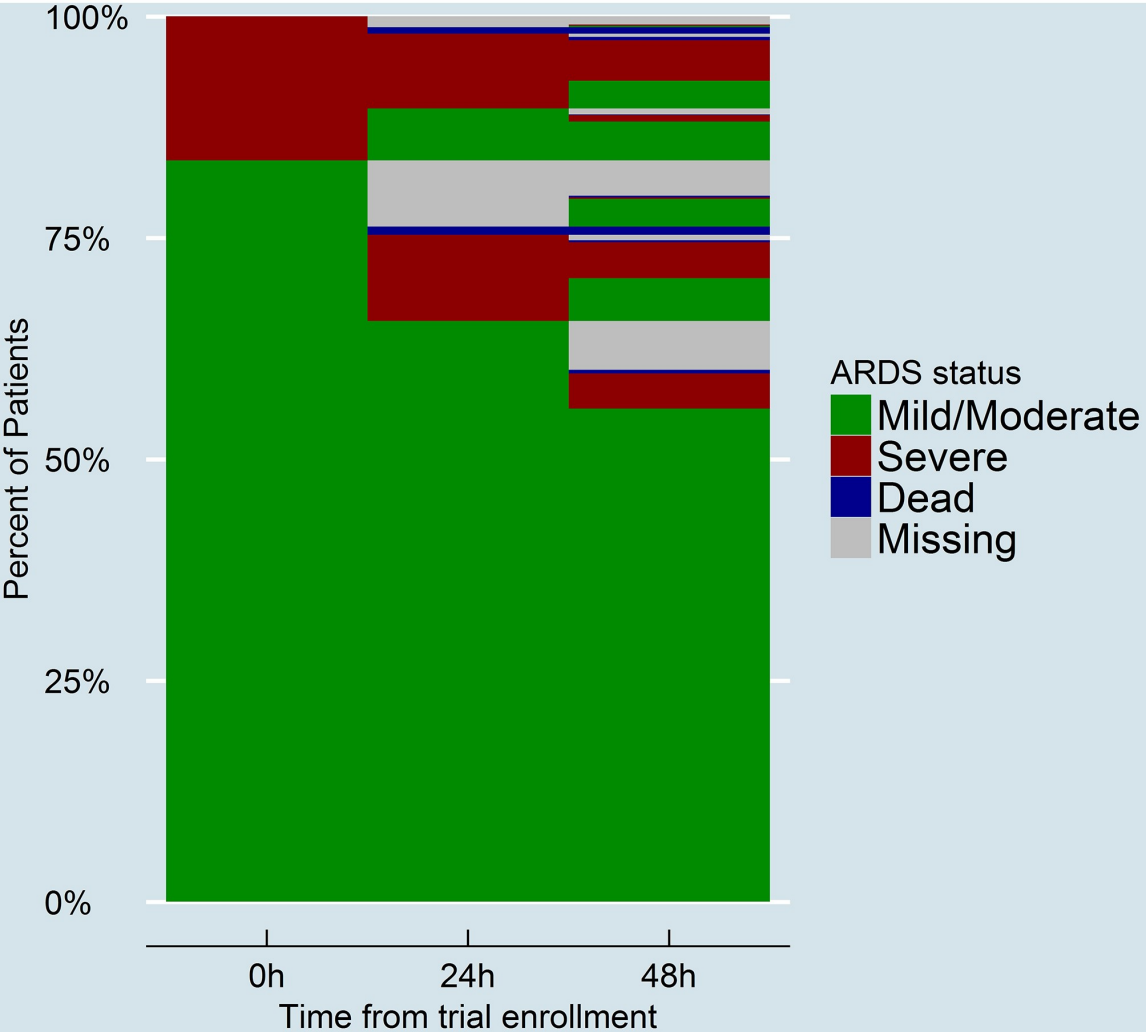
Lasagna plot depicting the trajectory of hypoxemia of each individual patient during the first two study days after trial enrollment. On the second study day, some patients that initially had mild/moderate ARDS (shown as green) progressed to persistent severe ARDS (shown in red). Some patients with an initial presentation of severe ARDS (red) continued to have severe ARDS on the second study day.

Of the 1531 unique patients with ARDS enrolled in the randomized controlled trials who were alive on the second study day and for whom relevant data were available [9–11], 232 (15%) met criteria for persistent severe ARDS. The trajectory of hypoxemia for patients during the first two study days after trial enrollment was presented as a lasagna plot (Fig 1).

Baseline characteristics are summarized in Table 1. Usage of vasopressors was greater [144/232 (62%) versus 623/1299 (48%); p<0.001] and Acute Physiology and Chronic Health Evaluation (APACHE III) scores (an assessment of baseline severity of illness) were higher [99 (81–119) versus 89 (70–107); p<0.001] in patients with versus without persistent severe ARDS. Patients with persistent severe ARDS were more likely to have severe hypoxemia at baseline compared to patients without persistent severe ARDS [132/232 (57%) versus 442/1299 (34%); p<0.001]. Interestingly, 100 (43%) of 232 of patients with persistent severe ARDS had mild or moderate hypoxemia at baseline. PaO_2_:FiO_2_ was lower in patients with versus without persistent severe ARDS [110 (86–141) versus 170 (128–226); p<0.001]. With regard to ventilator parameters, plateau pressure [28 (23–31) versus 23 (19–27); p<0.001] and positive end-expiratory pressure [12 (10–15) versus 9 (5–10); p<0.001] were greater in patients with versus without persistent severe ARDS.

**Table 1:** Baseline characteristics of patients with versus without persistent severe ARDS.

	With persistent severe ARDS^a^	Without persistent severe ARDS	p value
Number of patients	232 (15%)	1299 (85%)	
Age, years	49 (38–62)	53 (43–64)	0.014
Male sex	102 (44%)	689 (53%)	0.013
Race			0.886
White	181 (78%)	1031 (79%)	
Black	41 (18%)	213 (16%)	
Other	10 (4%)	55 (4%)	
Body mass index	29 (24–36)	28 (24–34)	0.049
Usage of vasopressors	144 (62%)	623 (48%)	<0.001
APACHE III score	99 (81–119)	89 (70–107)	<0.001
Primary risk factor of ARDS			
Pneumonia	164 (71%)	808 (62%)	0.016
Sepsis	40 (17%)	228 (18%)	0.983
Aspiration	10 (4%)	138 (11%)	0.004
Trauma	6 (3%)	54 (4%)	0.341
Multiple transfusions	2 (1%)	22 (2%)	0.564
Other	11 (5%)	53 (4%)	0.775
Non-pulmonary organ failure			
Circulatory	183 (79%)	896 (69%)	0.003
Coagulation	45 (20%)	223 (17%)	0.472
Hepatic	47 (21%)	161 (13%)	0.004
Renal	53 (23%)	306 (24%)	0.840
Severity of ARDS^b^			<0.001
Mild	23 (10%)	250 (19%)	
Moderate	77 (33%)	607 (47%)	
Severe	132 (57%)	442 (34%)	
PaO_2_:FiO_2_	110 (86–141)	170 (128–226)	<0.001
Change in PaO_2_:FiO_2_ from screening to enrollment	13 (-14-49)	36 (-4-90)	<0.001
Driving pressure	14 (11–19)	14 (11–17)	0.062
Plateau pressure	28 (23–31)	23 (19–27)	<0.001
Positive end-expiratory pressure	12 (10–15)	9 (5–10)	<0.001
Minute ventilation	11 (10–14)	11 (9–13)	<0.001

Abbreviations: ARDS, acute respiratory distress syndrome; APACHE, acute physiology and chronic health evaluation; PaO_2_:FiO_2_, partial pressure of arterial oxygen to fraction of inspired oxygen ratio. Data are presented as n (%) or median (interquartile range).

^a^ Persistent severe ARDS was defined by a PaO_2_:FiO_2_ of equal to or less than 100 mmHg on the second study day following trial enrollment.

^b^ Severity of ARDS at screening was categorized based on the Berlin definition.

Outcomes are summarized in Table 2. Mortality at 60 days was higher in patients with persistent severe ARDS compared to those without persistent severe ARDS [95/232 (41%) versus 233/1299 (18%); p<0.001]. Consistently, patients with persistent severe ARDS had fewer ventilator-free [0 (0–17) versus 22 (8–25); p<0.001], ICU-free [0 (0–14) versus 19 (7–23); p<0.001] and non-pulmonary organ failure-free [3 (0–21) versus 20 (1–26); p<0.001] days than comparators.

**Table 2:** Outcomes of patients with versus without persistent severe ARDS.

Outcome^a^	With persistent severe ARDS^b^ (n = 232)	Without persistent severe ARDS (n = 1299)	p value
60-day mortality	95 (41%)	233 (18%)	<0.001
Ventilator-free days	0 (0–17)	22 (8–25)	<0.001
ICU-free days	0 (0–14)	19 (7–23)	<0.001
Non-pulmonary organ failure-free days	3 (0–21)	20 (1–26)	<0.001

Abbreviations: ARDS, acute respiratory distress syndrome; ICU, intensive care unit. Data are presented as n (%) or median (interquartile range).

^a^ Patients discharged from hospital with unassisted breathing before 60 days considered to be alive at 60 days. Ventilator-free days, ICU-free days and non-pulmonary organ failure-free days were calculated by the number of days in the first 28 days that a patient was alive and not on a ventilator, not in the ICU, or free of non-pulmonary organ failure, respectively.

^b^ Persistent severe ARDS was defined by a partial pressure of arterial oxygen to fraction of inspired oxygen ratio (PaO_2_:FiO_2_) of equal to or less than 100 mmHg on the second study day following trial enrollment.

Within the individual trials included in our analysis (ALTA, EDEN and SAILS) [9–11], the estimate of treatment effect of the intervention (albuterol, feeding and statins, respectively) did not differ between patients with and without persistent severe ARDS (S1 Table).

A sensitivity analysis, in which persistent severe ARDS was defined as PaO_2_:FiO_2_ of equal to or less than 100 mmHg on the third study day instead of the second, is presented in S2 Table. The results of the sensitivity analysis corroborated those of the main analysis.

The characteristics of the predictive logistic regression model for persistent severe ARDS are shown in Table 3. PaO_2_:FiO_2_, FiO_2_, hepatic failure and positive end-expiratory pressure at enrollment were the selected variables to be included in the predictive model based on the machine learning techniques described in the Methods section. No multicollinearity among predictors was found. The AUC of the model for predicting persistent severe ARDS was 0.79 (95% CI 0.75–0.82) in the derivation dataset and 0.76 (95% CI 0.70–0.81) in the validation dataset. Of note, PaO_2_:FiO_2_, FiO_2_, hepatic failure and positive end-expiratory pressure at enrollment were also the included variables in a predictive logistic regression model to identify patients with mild or moderate hypoxemia at trial enrollment who deteriorated to severe hypoxemia on the second study day after trial enrollment (S3 Table and S1 Fig).

**Table 3:** Logistic regression model for predicting persistent severe ARDS using variables available at trial enrollment.

	Univariate analysis		Multivariate analysis	
	Odds Ratio (95% CI)	p value	Odds ratio (95% CI)	p value
PaO_2_:FiO_2_^a^	0.84 (0.81–0.88)	<0.001	0.90 (0.86–0.94)	<0.001
FiO_2_^b^	1.55 (1.42–1.69)	<0.001	1.17 (1.03–1.32)	0.014
Hepatic organ failure	2.16 (1.45–3.22)	<0.001	2.12 (1.35–3.32)	0.001
Positive end-expiratory pressure	1.17 (1.13–1.22)	<0.001	1.08 (1.03–1.13)	0.001

Abbreviations: ARDS, acute respiratory distress syndrome; CI, confidence intervals; PaO_2_:FiO_2_, partial pressure of arterial oxygen to fraction of inspired oxygen ratio.

^a^ Reported as per 10 point change.

^b^ Reported as per 10% change.

## Discussion

This secondary analysis of patient-level data from recent ARDSNet randomized trials demonstrates that patients with persistent severe ARDS had distinct baseline characteristics and worse clinical outcomes, including mortality and non-pulmonary organ failure-free days, when compared to patients without persistent severe ARDS. PaO_2_:FiO_2_, FiO_2_, hepatic failure and positive end-expiratory pressure at enrollment were useful variables to predict persistent severe ARDS.

We found that patients with persistent severe ARDS had distinct baseline characteristics, including more severe illness and more severe hypoxemia than comparators. Interestingly, almost half of patients with severe hypoxemia on the second study day after trial enrollment had mild or moderate hypoxemia initially, which deteriorated later. This reinforces the notion that hypoxemia alone (expressed as PaO_2_:FiO_2_), especially assessed as early as the time of diagnosis of ARDS, may be an insufficient predictor of the natural course of ARDS [15–17]. That said, three (PaO_2_:FiO_2_, FiO_2_, and positive end-expiratory pressure) out of the four variables comprising the predictive model were indices of oxygenation.

We found that patients with persistent severe ARDS had poorer clinical outcomes than those without persistent severe ARDS confirming our hypothesis that prognostic enrichment of ARDSNet trials would have been successful by selectively enrolling such patients. Indeed, persistent severe ARDS had a statistically significant and clinically meaningful association with 60-day mortality, ventilator-free days, ICU-free days and non-pulmonary organ failure-free days. Therefore, enrollment of such patients (who are likely to experience the abovementioned clinical outcomes) may be one way to build personalized medicine and provide help to the most needed patients. However, we should keep in mind that patients with persistent severe ARDS comprised only the 15% of the initial patient population, which means that targeting such patients would substantially prolong the enrollment period undermining the feasibility of trials.

On the other hand, predictive enrichment of ARDSNet trials (ALTA, EDEN and SAILS) would have not been successful by selectively enrolling patients with persistent severe ARDS [9–11]. Indeed, in each individual trial [9–11], we found no difference in terms of the treatment effect of the intervention between patients with versus without persistent severe ARDS. Two subphenotypes (an hyperinflammatory and another less inflammatory) of ARDS have been recently identified with differential response to interventions [15, 18], such as positive end-expiratory pressure and fluid administration. The lack of predictive enrichment in our model contradicted our hypothesis that selective enrollment of patients with persistent severe ARDS would identify a homogenous population with a common underlying histopathology of diffuse alveolar damage more likely to respond to ARDS therapies [7, 19]. Two conjectures could be made for this finding. Firstly, patients with persistent severe ARDS enrolled in the ARDSNet trials [9–11] might not have had diffuse alveolar damage. Although common, Thille *et al*. found some heterogeneity in this finding, with only 69% of the severe ARDS patients found to have diffuse alveolar damage after 72 hours [7]. There were no data available from open lung biopsies for these patients to explore whether this was indeed the case [9–11]. Secondly, patients with persistent severe ARDS enrolled in the ARDSNet trials [9–11] might indeed have had diffuse alveolar damage, but the tested interventions (namely, albuterol, feeding and statins) might not be effective against it. Diffuse alveolar damage, albeit presently considered as the histopathological correlate of ARDS [20], may be a nonspecific terminal feature present in various lung processes [21] making it a suboptimal target for pharmacological interventions.

By applying sophisticated machine learning techniques, we found that PaO_2_:FiO_2_, FiO_2_ and positive end-expiratory pressure at enrollment may be useful to predict persistent severe ARDS (i.e., PaO_2_:FiO_2_ of equal to or less than 100 mmHg on the second study day after trial enrollment). This finding renders support to previous work based on observational studies, which suggested that not only PaO_2_:FiO_2_ but also FiO_2_ and positive end-expiratory pressure should be taken into consideration [22]. Interestingly, PaO_2_:FiO_2_, FiO_2_ and positive end-expiratory pressure assessed at enrollment (i.e., under standardized ventilator settings) were also used to define inclusion criteria of the successful PROSEVA trial, which tested prone positioning in ARDS [23]. Another potential strength of our predictive model is that, unlike previous outcome scores [24], it does not focus only on mortality. Rather, we propose that a model to predict the composite outcome of mortality and persistent severe ARDS (instead of mortality alone) may be clinically useful given that both patients at risk of mortality and those at risk of refractory hypoxemia require early consideration by clinicians to initiate aggressive treatment.

Our study has limitations. Firstly, although we had access to extensive information for patients enrolled in high quality randomized controlled trials [9–11], data on PaO_2_:FiO_2_ the second day after trial enrollment were missing in one-sixth of patients. This is a common limitation of studies in this field [25]. Importantly, baseline characteristics and clinical outcomes of the latter patients were similar to those of included patients and it is therefore unlikely that their exclusion introduced any bias in our analyses. Secondly, the performance of our predictive model (with an AUC of around 0.8) was reasonable, but not great. This means that investigators may not be able to reliably identify who will suffer from persistent severe ARDS by using only clinical and physiological variables. Unfortunately, we could not determine whether inclusion of biomarkers would improve the performance of our predictive model because such data were not available for the three most recent ARDSNet trials [9–11]. Finally, one may argue that the three different trials (i.e., ALTA, EDEN and SAILS) [9–11] could have been used as a stratification factor when allocating patients either to derivation or validation cohort, and in the final logistic regression model. While the trials [9–11] were not explicitly used as a stratification factor, given the small number of trials relative to the number of patients, the balance was similar in the derivation (15%, 51%, 34% of ALTA, EDEN, and SAILS, respectively) and validation (14%, 50%, 36% of ALTA, EDEN, and SAILS, respectively) cohorts. Also, since we attempted to build a model suitable for any patient in any of these trials (or beyond), we did not use trial as a stratification factor in our final logistic regression model.

## Conclusions

In conclusion, this secondary analysis suggests that patients with persistent severe ARDS have distinct baseline characteristics and poor prognosis. Identifying such patients at trial enrollment may be useful for prognostic enrichment of trials of ARDS.

## Supporting information

S1 Table Treatment effects overall and within subgroups defined by persistent severe ARDS in each ARDSNet trial. (DOCX)

S2 Table Baseline characteristics and outcomes of patients with versus without persistent severe ARDS on third study day after trial enrollment (sensitivity analysis). (DOCX)

S3 Table Logistic regression model for predicting deterioration from mild or moderate ARDS at trial enrollment to severe ARDS on second study day after trial enrollment using variables available at trial enrollment. (DOCX)

S1 Fig Receiver operating curves for logistic regression predicting deterioration from mild or moderate acute respiratory distress syndrome (ARDS) at trial enrollment to severe ARDS on second study day after trial enrollment using variables available at the time of trial enrollment in the derivation (left panel) and validation (right panel) dataset. (DOCX)
