# Supplementary material for: Persistent severe acute respiratory distress syndrome for the prognostic enrichment of trials
Source: PLoS One. 2020 Jan 27;15(1):e0227346. doi: 10.1371/journal.pone.0227346 (PMC6984692; doi:10.1371/journal.pone.0227346)
Supplement: S1 Table — (DOCX) [file pone.0227346.s001.docx]

**S1 Table. Treatment effects overall and within subgroups defined by persistent severe ARDS in each ARDSNet trial.**

|  | **Treatment** | | **p value** | **Risk Ratio**  **(95% CI)** | **p value** |
| --- | --- | --- | --- | --- | --- |
| **ALTA** | **Placebo** | **Albuterol** |  |  |  |
| 60-day mortality | 18 (16%) | 26 (20%) | 0.507 | 1.23 (0.71-2.12) | 0.460 |
| psARDS | 3 (27%) | 5 (38%) | 0.679 | 1.41 (0.43-4.61) | 0.562 |
| without psARDS | 15 (15%) | 21 (18%) | 0.590 | 1.19 (0.65-2.19) | 0.574 |
| Ventilator-free Days | 22 (13-25) | 21 (0-25) | 0.223 |  |  |
| psARDS | 15 (3-20) | 9 (0-14) | 0.201 |  |  |
| without psARDS | 22 (13-25) | 21 (0-25) | 0.320 |  |  |
| ICU-free days | 19 (11-23) | 17 (1-22) | 0.059 |  |  |
| psARDS | 12 (2-16) | 1 (0-12) | 0.234 |  |  |
| without psARDS | 20 (11-23) | 18 (4-22) | 0.082 |  |  |
| Non-pulmonary organ failure-free days | 19 (10-24) | 18 (2-23) | 0.188 |  |  |
| psARDS | 11 (1-23) | 11 (0-19) | 0.857 |  |  |
| without psARDS | 19 (12-24) | 18 (2-24) | 0.199 |  |  |
| **EDEN** | **Full** | **Trophic** |  |  |  |
| 60-day mortality | 82 (21%) | 84 (22%) | 0.861 | 1.03 (0.79-1.35) | 0.832 |
| psARDS | 28 (36%) | 29 (48%) | 0.224 | 1.31 (0.88-1.94) | 0.185 |
| without psARDS | 54 (17%) | 55 (17%) | 0.917 | 0.98 (0.69-1.37) | 0.885 |
| Ventilator-free days | 19 (0-24) | 20 (0-24) | 0.504 |  |  |
| psARDS | 0 (0-17) | 0 (0-12) | 0.125 |  |  |
| without psARDS | 21 (8-25) | 22 (8-25) | 0.554 |  |  |
| ICU-free days | 17 (1-22) | 17 (2-22) | 0.666 |  |  |
| psARDS | 0 (0-16) | 0 (0-5) | 0.164 |  |  |
| without psARDS | 19 (8-23) | 19 (7-23) | 0.706 |  |  |
| Non-pulmonary organ failure-free days | 18 (1-25) | 18 (1-26) | 0.452 |  |  |
| psARDS | 4 (0-23) | 2 (0-16) | 0.226 |  |  |
| without psARDS | 20 (2-26) | 21 (3-26) | 0.362 |  |  |
| **SAILS** | **Placebo** | **Rosuvastatin** |  |  |  |
| 60-day mortality | 55 (21%) | 63 (25%) | 0.344 | 1.18 (0.86-1.62) | 0.311 |
| psARDS | 12 (36%) | 18 (49%) | 0.341 | 1.34 (0.76-2.34) | 0.299 |
| without psARDS | 43 (19%) | 45 (21%) | 0.635 | 1.10 (0.76-1.60) | 0.614 |
| Ventilator-free days | 20 (0-25) | 20 (0-25) | 0.952 |  |  |
| psARDS | 0 (0-20) | 0 (0-13) | 0.279 |  |  |
| without psARDS | 21 (4-25) | 22 (12-26) | 0.644 |  |  |
| ICU-free days | 17 (1-23) | 17 (1-23) | 0.953 |  |  |
| psARDS | 0 (0-18) | 0 (0-7) | 0.111 |  |  |
| without psARDS | 19 (5-23) | 19 (9-24) | 0.530 |  |  |
| Non-pulmonary organ failure-free days | 20 (0-26) | 18 (0-26) | 0.357 |  |  |
| psARDS | 4 (0-20) | 0 (0-23) | 0.221 |  |  |
| without psARDS | 21 (1-26) | 21 (0-26) | 0.664 |  |  |

Abbreviations: ARDS, acute respiratory distress syndrome; CI, confidence intervals; psARDS, persistent severe ARDS; ICU, intensive care unit.

Data are presented as n (%) or median (interquartile range).

Patients discharged from hospital with unassisted breathing before 60 days considered to be alive at 60 days. Ventilator-free days, ICU-free days and non-pulmonary organ failure-free days were calculated by the number of days in the first 28 days that a patient was alive and not on a ventilator, not in the ICU, or free of non-pulmonary organ failure, respectively.
