# Supplementary material for: Persistent severe acute respiratory distress syndrome for the prognostic enrichment of trials
Source: PLoS One. 2020 Jan 27;15(1):e0227346. doi: 10.1371/journal.pone.0227346 (PMC6984692; doi:10.1371/journal.pone.0227346)
Supplement: S2 Table — (DOCX) [file pone.0227346.s002.docx]

**S2 Table. Baseline characteristics and outcomes of patients with versus without persistent severe ARDS on third study day after trial enrollment (sensitivity analysis).**

|  | **With persistent**  **severe ARDS** | **Without persistent**  **severe ARDS** | **p value** |
| --- | --- | --- | --- |
| Number of patients | 184 | 1338 |  |
| Age, years | 49 (39-62) | 53 (42-64) | 0.047 |
| Male sex | 80 (44%) | 703 (53%) | 0.026 |
| Race |  |  | 0.669 |
| White | 150 (82%) | 1057 (79%) |  |
| Black | 26 (14%) | 224 (17%) |  |
| Other | 8 (4%) | 57 (4%) |  |
| Body mass index | 30 (25-37) | 28 (24-34) | 0.006 |
| Usage of vasopressors | 106 (58%) | 643 (48%) | 0.018 |
| APACHE III score | 100 (81-122) | 88 (70-107) | <0.001 |
| Primary risk factor of ARDS |  |  |  |
| Pneumonia | 126 (69%) | 841 (63%) | 0.160 |
| Sepsis | 34 (19%) | 238 (18%) | 0.899 |
| Aspiration | 11 (6%) | 133 (10%) | 0.112 |
| Trauma | 3 (2%) | 59 (4%) | 0.112 |
| Multiple Transfusions | 2 (1%) | 17 (1%) | 1.000 |
| Other | 9 (5%) | 54 (4%) | 0.727 |
| Non-Pulmonary Organ Failure |  |  |  |
| Circulatory | 141 (77%) | 928 (69%) | 0.053 |
| Coagulation | 31 (17%) | 225 (17%) | 1.000 |
| Hepatic | 34 (19%) | 167 (13%) | 0.039 |
| Renal | 41 (23%) | 312 (24%) | 0.837 |
| Severity of ARDS* |  |  | <0.001 |
| Mild | 21 (11%) | 247 (19%) |  |
| Moderate | 63 (34%) | 613 (46%) |  |
| Severe | 100 (54%) | 478 (36%) |  |
| PaO_2_:FiO_2_ | 108 (83-140) | 168 (127-224) | <0.001 |
| Change in PaO_2_:FiO_2_ from screening to enrollment | 12 (-24-43) | 37 (-1-90) | <0.001 |
| Driving pressure | 14 (11-19) | 14 (11-17) | 0.178 |
| Plateau pressure | 27 (23-31) | 23 (19-27) | <0.001 |
| Positive end-expiratory pressure | 12 (10-15) | 8 (5-10) | <0.001 |
| Minute ventilation | 11.2 (9.45-14.0) | 10.5 (8.5-12.7) | <0.001 |
| 60-day mortality | 73 (40%) | 238 (18%) | <0.001 |
| Ventilator-free days | 0 (0-13) | 22 (10-25) | <0.001 |
| ICU-free days | 0 (0-10) | 19 (8-23) | <0.001 |
| Non-pulmonary  organ failure-free days | 3 (0-20) | 21 (1-26) | <0.001 |

Abbreviations: ARDS, acute respiratory distress syndrome; APACHE, acute physiology and chronic health evaluation; PaO_2_:FiO_2_, partial pressure of arterial oxygen to fraction of inspired oxygen ratio; ICU, intensive care unit.

Data are presented as n (%) or median (interquartile range).

*Severity of ARDS at screening was categorized based on the Berlin definition.

Patients discharged from hospital with unassisted breathing before 60 days considered to be alive at 60 days. Ventilator-free days, ICU-free days and non-pulmonary organ failure-free days were calculated by the number of days in the first 28 days that a patient was alive and not on a ventilator, not in the ICU, or free of non-pulmonary organ failure, respectively.

Persistent severe ARDS was defined by a PaO_2_:FiO_2_ of equal to or less than 100 mmHg on third study day following trial enrollment.
