# Supplementary material for: Persistent severe acute respiratory distress syndrome for the prognostic enrichment of trials
Source: PLoS One. 2020 Jan 27;15(1):e0227346. doi: 10.1371/journal.pone.0227346 (PMC6984692; doi:10.1371/journal.pone.0227346)
Supplement: S3 Table — (DOCX) [file pone.0227346.s003.docx]

**S3 Table. Logistic regression model for predicting deterioration from mild or moderate ARDS at trial enrollment to severe ARDS on second study day after trial enrollment using variables available at trial enrollment.**

|  | **Univariate analysis** | | **Multivariate analysis** | |
| --- | --- | --- | --- | --- |
|  | **Odds Ratio (95% CI)** | **p value** | **Odds ratio (95% CI)** | **p value** |
| PaO_2_:FiO_2_* | 0.86 (0.82-0.90) | <0.001 | 0.90 (0.85-0.94) | <0.001 |
| FiO_2_** | 1.49 (1.33-1.67) | <0.001 | 1.18 (1.02-1.37) | 0.031 |
| Hepatic organ failure | 2.54 (1.57-4.09) | 0.001 | 2.58 (1.54-4.35) | <0.001 |
| Positive end-expiratory pressure | 1.15 (1.09-1.20) | <0.001 | 1.08 (1.02-1.14) | 0.009 |

Abbreviations: ARDS, acute respiratory distress syndrome; CI, confidence intervals; PaO_2_:FiO_2_, partial pressure of arterial oxygen to fraction of inspired oxygen ratio.

*Reported as per 10 point change.

**Reported as per 10% change.

Persistent severe ARDS was defined by PaO_2_:FiO_2_ of equal to or less than 100 mmHg on second study day following trial enrollment.
