# Supplementary material for: Persistent severe acute respiratory distress syndrome for the prognostic enrichment of trials
Source: PLoS One. 2020 Jan 27;15(1):e0227346. doi: 10.1371/journal.pone.0227346 (PMC6984692; doi:10.1371/journal.pone.0227346)
Supplement: S1 Fig — (DOCX) [file pone.0227346.s004.docx]

**S1 Figure. Receiver operating curves for logistic regression predicting deterioration from mild or moderate acute respiratory distress syndrome (ARDS) at trial enrollment to severe ARDS on second study day after trial enrollment using variables available at the time of trial enrollment in the derivation (left panel) and validation (right panel) dataset.** Red circle indicates Youden’s optimal cut point in the derivation dataset. 95% confidence intervals are shown. Abbreviations: AUC, area under the receiver operating curve; NPV, negative predictive value; PPV, positive predictive value; Spec, specificity; Sens, sensitivity.

**
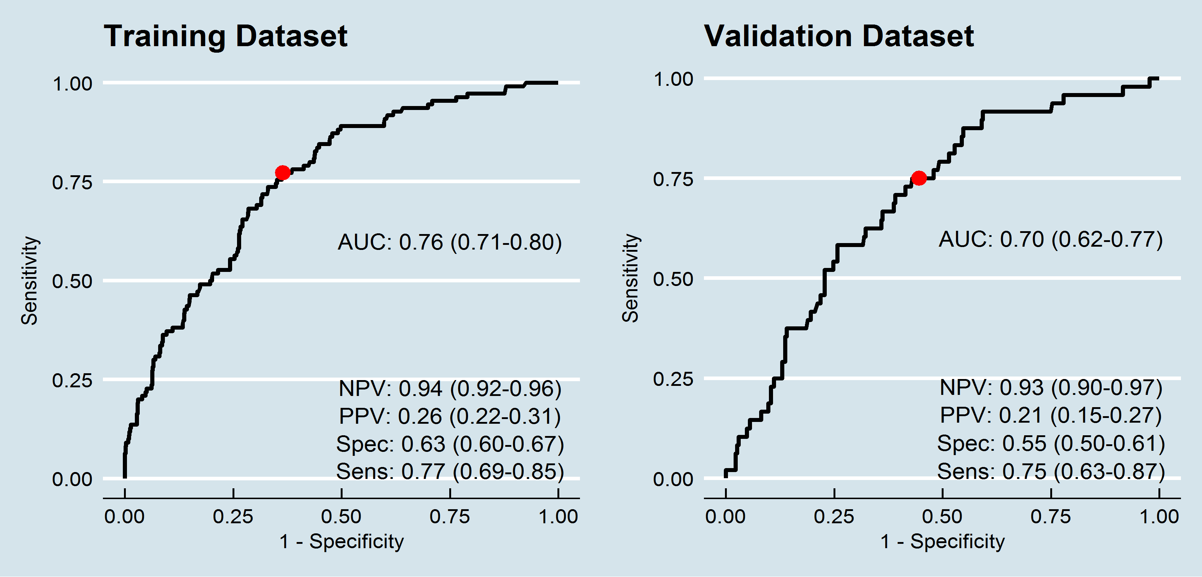
**
